# A Chaotic Cryptosystem for Images Based on Henon and Arnold Cat Map

**DOI:** 10.1155/2014/536930

**Published:** 2014-08-28

**Authors:** Ali Soleymani, Md Jan Nordin, Elankovan Sundararajan

**Affiliations:** ^1^Software Technology and Management Center (Softam), Faculty of Information Science and Technology, Universiti Kebangsaan Malaysia, 43600 Bangi, Selangor, Malaysia; ^2^Center for Artificial Intelligence Technology (CAIT), Faculty of Information Science and Technology, Universiti Kebangsaan Malaysia, 43600 Bangi, Selangor, Malaysia

## Abstract

The rapid evolution of imaging and communication technologies has transformed images into a widespread data type. Different types of data, such as personal medical information, official correspondence, or governmental and military documents, are saved and transmitted in the form of images over public networks. Hence, a fast and secure cryptosystem is needed for high-resolution images. In this paper, a novel encryption scheme is presented for securing images based on Arnold cat and Henon chaotic maps. The scheme uses Arnold cat map for bit- and pixel-level permutations on plain and secret images, while Henon map creates secret images and specific parameters for the permutations. Both the encryption and decryption processes are explained, formulated, and graphically presented. The results of security analysis of five different images demonstrate the strength of the proposed cryptosystem against statistical, brute force and differential attacks. The evaluated running time for both encryption and decryption processes guarantee that the cryptosystem can work effectively in real-time applications.

## 1. Introduction

Some researchers utilized conventional cryptosystems to directly encrypting images. But this is not advisable due to large data size and real-time constraints of image data. Conventional cryptosystems require a lot of time to directly encrypt thousands of image pixels value. On the other hand, unlike textual data, a decrypted image is usually acceptable even if it contains small levels of distortion. For all the above mentioned reasons, the algorithms that function well for textual data may not be suitable for multimedia data [1]. Many studies have been performed on the use of textual encryption algorithms for images by modifying the algorithms to adapt with image characteristics. One such option for encrypting an image is to consider a 2D array of image pixels value as a 1D data stream and to then encrypt this stream with any conventional cryptosystem [2, 3]. This would be considered a naïve approach and usually is suitable for text and occasionally for small images files that are to be transmitted over a fleet dedicated channel [4]. Subramanyan et al. [5] proposed an image encryption algorithm based on AES-128 in which the encryption process is a bitwise XOR operation on a set of image pixels. This method employs an initial 128-bit key and an AES key expansion process that changes the key for every set of pixels. The secret keys are generated independently at both the sender and the receiver sides based on the AES key expansion process. Therefore, the initial key alone is shared rather than the whole set of keys.

## 2. Chaos and Cryptography

Chaotic maps are simple functions and are iterated quickly. Chaos-based image encryption systems are therefore fast enough for real-time applications. Chaos is a natural phenomenon discovered by Edward Lorenz in 1963 while studying the butterfly effect in dynamical systems. The butterfly effect describes the sensitivity of a system to initial conditions as mentioned in Lorenz's paper titled “Does the Flap of a Butterfly's Wings in Brazil set off a Tornado in Texas?” [6]. The flapping wings represent a tiny variation in the initial conditions of the dynamic system that causes a chain of events leading to large-scale changes in the future. Had the butterfly not flapped its wings, the trajectory of the system might have been vastly different [7]. In general, this means that a small variance in the initial parameters (even in ten-millionth place value) could yield widely divergent results. Hence, for a chaotic system, rendering long-term prediction is impossible in general. This means that having initial conditions of these systems makes their future behavior predictable. This behavior, which derived from a natural phenomenon, is known as deterministic chaos or, simply, chaos and exhibits by chaotic maps. Such maps are classified as continuous maps and discrete maps.

In the 1990s, numerous researchers found that there are some relationships between properties that have counterparts in chaos and cryptography. A high sensitivity to initial conditions, with deterministic pseudorandom behavior, is an interesting similarity between chaotic maps and cryptographic algorithms. Furthermore, confusion and diffusion are two general principles in the design of cryptography algorithms that lead to the concealing of the statistical structure of pixels in a plain image and to a decrease in the statistical dependence of a plain image and the corresponding encrypted one. Applying a mixing property on chaos-based encryption algorithms will increase the complexity of the cipher image.

Chaotic maps are assigned to discrete and continuous time-domains. Discrete maps are usually in the form of iterated functions, which corresponded to rounds in cryptosystems. This similarity between cryptography and discrete chaotic dynamic systems is utilized to propose chaotic cryptosystems. Each map has some parameters that are equivalent to the encryption keys in cryptography. In stream cipher, a chaotic system is applied to generate a pseudorandom key stream but in block ciphers, the plaintext or the secret key(s) are used as the initial and control parameters. Finally, some iteration is applied on the chaotic systems to obtain the cipher-text. Security and complexity are significant concerns in cryptosystems. These should be considered when selecting a map and its parameters for use in cryptography [8].

### 2.1. Related Works

The first chaos-based cryptosystem was proposed by Matthews in 1989 [29]. Subsequently, the amount of research on chaotic cryptography increased rapidly, while trying to break (and find the weakness of) the proposed schemes in order to improve chaos-based cryptosystems.

The algorithm proposed by Wang et al. [30] for encrypting color images utilizing a logistic map was broken by Li et al. [31]. Another cryptosystem analyzed by Li et al. [32] is the recent work of Zhu [26]. Zhu applied hyperchaotic sequences to generate the key stream but Li in his work proved that the proposed algorithm was not sufficiently robust against a chosen plaintext attack. Another weak cryptosystem is the combination of the Lorenz map and perceptron model of the neural network proposed by Wang et al. [33]. This chaotic algorithm was cracked by Zhang et al. [34] after analyzing its security by simulated attacks. The experimental results show that the secret key can be reconstructed after one pair of known-plaintext/ciphertext attacks. Furthermore, the effect of changing one bit in the plain image is a change in only one bit at the same position in an encrypted image. This is another weakness of Wang's proposed algorithm.

Many similar works have failed in security analysis. Hence, when designing and implementing a chaos-based cryptographic system, some important requirements should be kept in mind. A common framework was proposed by Alvarez and Li [35] for chaos-based cryptosystem designers. Implementation rules, key management tips, and security analysis approaches are three main issues suggested in their work. Adhering to these basic guidelines guarantees an acceptable level of security with the chaos-based cryptosystem scheme. Moreover, Alvarez and Li in [36] established a practical security analysis of a cryptosystem based on the Baker map [37]. In addition to breaking this cryptosystem due to vulnerability of the key, some countermeasures are introduced for improving and enhancing the security of similar cryptosystems. Alvarez and Li in another cryptanalysis work [38] presented that the nonlinear chaotic algorithm by Gao et al. [39] is insecure according to failure in the plaintext attack and statistical and key space analysis.

Chaos-based encryption algorithms are based on diverse types of chaotic maps and also on discrete maps. Most of these are a combination of two or more chaotic maps to achieve a greater level of complexity, security, and expanded key space. A combination of the Arnold cat map and the Chen map was the work of Guan et al. [40]. The Arnold cat map was applied to clutter the position of the pixels followed by XOR with the discrete output signal of the Chen map to modify the gray value of the cluttered pixels. This was analyzed and improved by Xiao et al. [41]. They found the weakness of the proposed algorithm and overcame the flaws.

To overcome the disadvantages of permutation-only cryptosystems, Fu et al. [12] proposed a novel shuffling algorithm which performs an efficient bit-level permutation in two stages of chaotic sequence sorting and Arnold cat map. Their analysis results show that this scheme is more secure and has much lower computational complexity than previous similar works.

Xu et al. [10] analyzed the improved work of Xiang et al. [42] and found two drawbacks. In their proposed letter, iterating Chen chaotic system generates random number sequence, which is more random in comparison with the sequence that was generated by logistic map in [42]. The second drawback is overcome by setting the parameter of Chen map using the last one byte of encrypted plaintext after every iteration that leads to a higher sensitivity of encrypted image to the plain one. This scheme is fast and secure according to simulation results and large size of key space, respectively.

To overcome the drawback of time-consuming real number arithmetic calculations in chaos-based image encryption techniques, a block cipher cryptosystem was proposed by Fouda et al. [27]. This fast and secure chaotic scheme is based on sorting the integer coefficients of linear diophantine equation (LDE), which is generated dynamically by only two rounds of any chaos map.

The scheme of Chen et al. [28] is another work that is proposed to enhance the efficiency of chaos-based encryption. They found that permutation-diffusion encryption approaches are produce with high computation of at least two chaotic maps and weak against known/chosen plaintext attacks. Hence, they proposed a dynamic mechanism to generate the state variables from the 3D or hyperchaotic maps for snake-like diffusion and pixel-swapping confusion. A tiny change (e.g., one pixel) will make a totally different key stream sequence at the first round of encryption.

Table 1 is a brief overview of some chaotic maps applied in image encryption. The Arnold cat map is the most commonly used map in chaos-based image encryption works with the main purpose of shuffling pixels of an image in a pseudorandom order.

### 2.2. Henon Map

Henon is a two-dimensional dynamic system proposed [43] to simplify the Lorenz map [44] with the same properties and is defined by (1). This might be easier to implement than the differential equations of the Lorenz system. Consider

(1) xi+1=yi+1+1−αxi2,yi+1=βxi.

**Figure 1:**
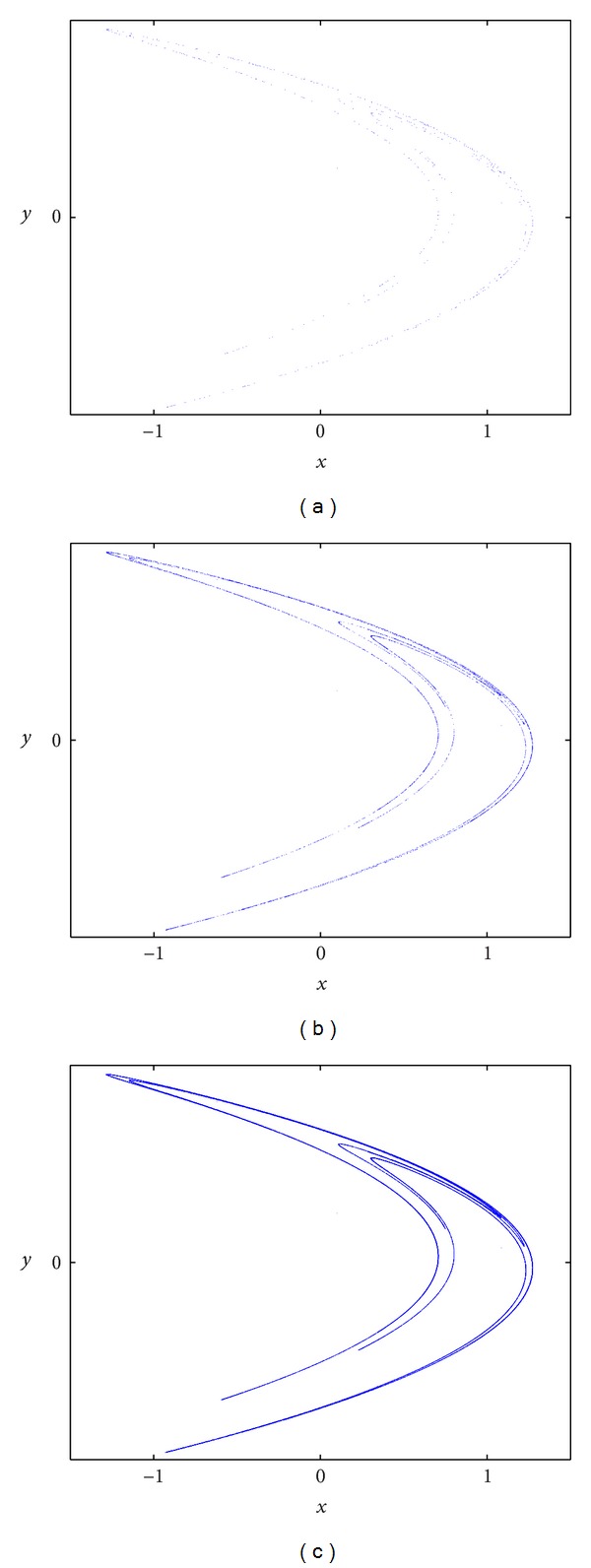
Henon map attractor after (a) 500, (b) 5000, and (c) 50000 iterations with initial parameters *α* = 1.4 and *β* = 0.3 and initial point (0.1, 0.1).

The initial parameters are *α*, *β* and the initial point is (*x*_0_, *y*_0_). Each point (*x*_*n*_, *y*_*n*_) is mapped to a new point (*x*_*n*+1_, *y*_*n*+1_) through the Henon map. For *α* = 1.4 and *β* = 0.3, the Henon function has chaotic behavior and the iterations have a boomerang-shaped chaotic attractor.  Figure 1 is the outline on a two-dimensional plane for the Henon map obtained from a distinct number of iterations starting from the chosen initial point (0.1, 0.1). Minute variations in the initial point will lead to major changes and different behavior.

### 2.3. Arnold Cat Map

ACM is a mixing discrete ergodic system that performs an area preserving stretch and fold mapping discovered by V. Arnold in 1968 using the image of a cat. This 2D transformation is based on a matrix with a determinant of 1 that makes this transformation reversible and described as

(2) $$\begin{array}{c} \Gamma :\;\left[\begin{array}{c} x^{\prime }\\ y^{\prime } \end{array}\right]=\left[\begin{array}{cc} 1 & P\\ Q & PQ+1 \end{array}\right]\left[\begin{array}{c} x\\ y \end{array}\right]\mathrm{mod}n. \end{array}$$

Here, *P* and *Q* are integers and (*x*, *y*) is the original position that is mapped to the new position (*x*′, *y*′). This transformation randomizes the original order of pixels or bits in an image. However, after sufficient iterations, the original image is reconstructed. Reverse mapping using (3) is a phase in decryption process to transform the shuffled image into the input image. The number of iterations in the permutation step must be equal to that of the reverse transformation. Consider

(3) $$\begin{array}{c} \Gamma^{\prime }:\left[\begin{array}{c} x\\ y \end{array}\right]=\left[\begin{array}{cc} PQ+1 & -P\\ -Q & 1 \end{array}\right]\left[\begin{array}{c} x^{\prime }\\ y^{\prime } \end{array}\right]\mathrm{mod}n. \end{array}$$

## 3. Proposed Cryptosystem Model

### 3.1. Initializing Prerequisite Values

**Figure 2:**
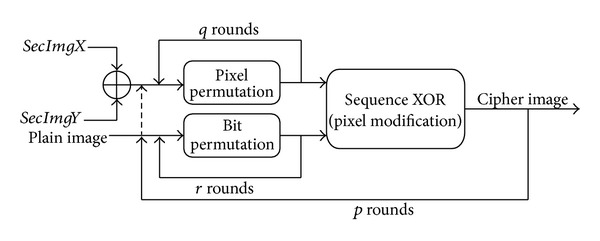
Proposed encryption scheme architecture.

In addition to *α*, *β*, and initial point (*x*_0_, *y*_0_) in (1), there are some other variables that must be initialized before running the algorithm. The proposed encryption architecture is shown in Figure 2. This scheme is based on two secret images and permutation steps in the bit level and pixel level. In the bitwise permutation, the pixel values are distorted but, in the pixel permutation, the pixels are shuffled without any alteration in value and histogram.

Creating the secret images and a set of parameters *P* and *Q* for the Arnold cat map are prerequisites for the encryption and decryption processes. Secret images have pseudo-random-like gray pixel distributions and are created using coordinates *x* and *y* generated by a Henon map. The secret images are the same as the plain image in height and width; therefore, the number of iterations for the Henon map depends on the total pixels in the plain image. In this work, experiments are performed on *m* × *m* gray-level images. Hence, the minimum iterations of Henon map should be *m* ^2^. The first few iterations seem fairly close together. Therefore, the total number of iterations is *m* ^2^ + 100, but the first 100 points are discarded to achieve higher randomness. Secret image pixels are generated using (4) and (6). The pix*X* and pix*Y* are sets of pseudorandom numbers (0 ≤ pix*X*_*i*_, pix*Y*_*i*_ ≤ 255) created by *x*-coordinates and *y*-coordinates of the Henon map and are considered pixel values. To shape the one-dimensional pixel values into an image, (5) and (7) are applied to create the 2D* secImgX* and* secImgY* secret image. The final secret image is generated by a combination of* secImgX* and* secImgY* by performing the XOR operation on the corresponding pixels as described by (8). Consider

(4) pixXi=abs(⌊x100+i×  γ⌋)mod⁡256, i=1,…,m2,

(5) $$\begin{array}{c} secImgX=\text{reshape}\left(\text{pix}X,m,m\right), \end{array}$$

(6) $$\begin{array}{c} {\text{pix}Y}_{j}=\text{abs}\left(\left\lfloor x_{100+i}\times \;\lambda \right\rfloor \right)\mathrm{mod}256,\;j=1,\ldots {,m}^{2}, \end{array}$$

(7) secImgY=reshape(pixY,m,m),

(8) secImg=xor(secImgX,secImgY).

The permutation steps by the Arnold cat map are based on the parameters *P* and *Q*. The *x*-coordinates and *y*-coordinates that result from the iterations of the Henon map are applied to generate parameters for the ACM. The pixels or bits of an input image that are permuted by the Arnold cat map return to its preliminary position after finite iterations. Attackers may be able to restore the original image by using this periodicity. To avoid such reconstruction of the input image, iteration is repeated for *q* rounds with different values for the parameters *P* and *Q* in each round. Equations (9) and (10) generate parameter values for the Arnold cat map. The number of generated parameters is equal to the total number of permutation rounds:

(9) Pi=abs(⌊x100+i×1014⌋)mod⁡δ, i=1,…,p∗(q+r),

(10) Qi=abs(⌊y100+i×1014⌋)mod⁡ϑ, i=1,…,p∗(q+r).

### 3.2. Encryption Process

Figure 2 presents the architecture of the proposed encryption scheme. This scheme has three inputs and three main functions, and the final result is the encrypted image. The plain image,* secImgX, *and* secImgY *are the three main inputs for this model. The primary functions are bit permutation, pixel permutation, and pixel modification. As illustrated in Figure 2, at the first step,* secImgX *and* secImgY *are XORed pixel by pixel to generate* secImg*. Then, pixels of the secret image are permuted *q* rounds. A simultaneous step is *r* rounds of bit-level permutation of the plain image. The outputs of these two phases are applied to pixel modification, which is a sequence XOR of consecutive pixels. The result is fed back to the bit permutation function (instead of the plain image) for additional *p* − 1 rounds while the* secImg *is permuted with new parameters at each round. The functions details are described in the following sections.

#### 3.2.1. Bit Permutation

**Figure 3:**
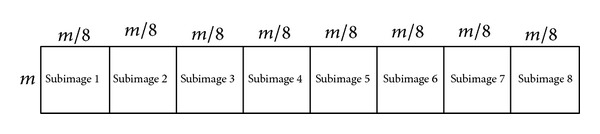
Splitting an image to 8 subimages.

For a gray-level image with a size of *m* × *m* pixels, the total bits are *m* × *m* × 8. Prior to bit permutation, the input image is divided into eight subimages. Each subimage is *m* × *m*/8 pixels or *m* × *m* bits in height and width as shown in Figure 3. Matrix (11) shows how the *k*th subimage is created. Replacing the corresponding pixel values of the input image at the proper position of the matrix would create the subimage. A pixel in the position (*i*, *j*) is an 8-bit value in the form of (12), where *b*(8) is the most significant bit (MSB) and *b*(1) is the least significant bit (LSB) of the pixel value in binary form. Every pixel value of the subimage converts to its binary format and creates the bit-plane. The bit-plane is a matrix with *m* rows and *m* columns and each element is one bit 0 or 1. Matrix (13) shows how to create the matrix for the bit-plane. Each subimage is converted to the equivalent *m* × *m* bit-plane and each bit-plane is permuted separately and independently:

(11) subImagek=[img(1,(k−1)∗m8+1)⋯img(1,k∗m8)img(2,(k−1)∗m8+1)⋯img(2,k∗m8)⋮⋮img(m,(k−1)∗m8+1)⋯  img(m,k∗m8)], k=1,…,8,

(12) img(i,j)=b(8)b(7)⋯b(1),

(13) bitPlanek=[b1,(k−1)∗(m/8+1)(8)  b1,(k−1)∗(m/8+1)(7)⋯b1,k∗(m/8)(1)b2,(k−1)∗(m/8+1)(8)b2,(k−1)∗(m/8+1)(7)⋯b1,k∗(m/8)(1)⋮⋮bm,(k−1)∗(m/8+1)(8)bm,(k−1)∗(m/8+1)(7)⋯bm,k∗(m/8)(1)].

After creating the* bitPlane* matrices, the Arnold cat map was applied to these matrices to permute the bits. In the permutation phase, the new location of each bit is calculated by (14). The pair of (*x*′, *y*′) is the new position of (*x*, *y*). At the first phase, the input image is permuted *r* times with different parameters *P* and *Q* where *i* = 1,…, *r*. Consider

(14) $$\begin{array}{c} \left[\begin{array}{c} x^{\prime }\\ y^{\prime } \end{array}\right]=\left[\begin{array}{cc} 1 & P_{i}\\ Q_{i} & P_{i}Q_{i}+1 \end{array}\right]\left[\begin{array}{c} x\\ y \end{array}\right]\mathrm{mod}m. \end{array}$$

After finishing this phase, the* bitPlane* matrices are changed to decimal values to reconstruct the image pixels.

#### 3.2.2. Pixel Permutation

Concurrent with bit permutation of the plain image, the secret image is permuted for *q* rounds at the pixel level to change the position of the pixels in a random manner. In contrast to bit permutation, pixel permutation does not affect the pixel values. Therefore, histograms of the plain image and the shuffled image are entirely the same. The permutation is performed *q* times with different parameters *P* and *Q* with *j* = 1,…, *q*. Consider

(15) $$\begin{array}{c} \left[\begin{array}{c} x^{\prime }\\ y^{\prime } \end{array}\right]=\left[\begin{array}{cc} 1 & P_{j}\\ Q_{j} & P_{j}Q_{j}+1 \end{array}\right]\left[\begin{array}{c} x\\ y \end{array}\right]\mathrm{mod}m. \end{array}$$

#### 3.2.3. Pixel Modification

The concluding phase is a sequence XOR to modify the pixel values. This step will cause extreme changes in the pixels of cipher image with even one bit change in a pixel in plain image. This step is based on the shuffled secret image and the bit-level permuted plain image. Equations (16) and (17) are used to change pixels consecutively. The output of this step is a modified image. For more confusion and modification, this step will repeat for *p* rounds. After each round, the result is replaced with a plain image, as input, and the secret image will permute *q* rounds. After completing *p* rounds, the final output is the cipher image. Consider

(16) img(1)=xor(img(1),secImg(1)),

(17) img(i)=xor(img(i−1),img(i),secImg(i)).

### 3.3. Decryption Process

**Figure 4:**
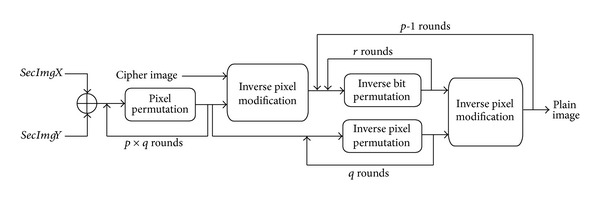
Decryption model architecture.

Figure 4 shows the decryption process. On the receiving side, the secret image is generated by XORing* secImgX *and* secImgY, *which are recreated using private parameters. Inverse pixel modification is performed on the cipher image and the secret image after *p* × *q* rounds of pixel permutations. The result of the secret image pixel permutation in the first step is saved for subsequent steps and at each round this is inverse permuted for *q* rounds and applied as an input to the inverse pixel modification function. The additional input is (the feedback of) the output of the inverse pixel modification function after *r* rounds of inverse bit permutation.

## 4. Experiments and Security Analysis

Experiments are carried out to analyze the proposed algorithm and evaluate their security and robustness. A vigorous encryption algorithm is resistant to attacks by an opponent or to unauthorized access. Due to the various types of attacks, a comprehensive security analysis is inevitable. This section analyzes the results of simulated attacks such as a statistical attack, a differential attack, a brute-force attack, and a known/chosen plaintext attack to demonstrate the strength of the proposed technique. Key space, encryption time, and decryption time are additional parameters that will affect the decision-making regarding the choice of applied cryptosystem. The selected images for the experiments are “Peppers,” “Baboon,” and “Fingerprint,” which are 512 × 512 gray images. “Cameraman” and “Chess-plate” are two additional images of size 256 × 256. The proposed algorithm is implemented using the MATLAB programming language on a PC with a 64-bit OS, Core i5 CPU, and 8 GB installed RAM.

### 4.1. Initial Values

The Henon map is the main function in this cryptosystem and the generated chaotic sequence is employed to produce both the secret image and the parameters for the Arnold cat map. Its initial value is one of the secret keys in this scheme. The chosen point (1.210000001, 0.360000001) is the starting point for generating the Henon chaotic sequence. *α* and *β* have fixed values of 1.4 and 0.3, respectively. As mentioned above, the test images sizes are 512 × 512 and 256 × 256 pixels. The number of iterations for the Henon map is based on the image size. The larger image has a total of 262144 pixels. The Henon map (18) is iterated 262244 times, but the first 100 points are discarded. Consider

(18) xi+1=yi+1+1−1.4xi2yi+1=0.3xi(x0=1.21000001,y0=0.36000001), i=0,…,262243.

Having obtained the set of *x* and *y*, we can create pixels of* secImgX* and* secImgY* by setting the values *γ* = 12345678 and *λ* = 87654321 in (4) and (6) as shown in (19) and (21), respectively. Then, we reshape them to form an image with the same size as the plain image using (20) and (22). The final secret image is the result of (23). Consider

(19) pixXi=abs(⌊x100+i×  12345678⌋)mod⁡256,i=1,…,262144,

(20) $$\begin{array}{c} secImgX=\text{reshape}\left(\text{pix}X,512,512\right), \end{array}$$

(21) pixYj=abs(⌊x100+i×  87654321⌋)mod⁡256,j=1,…,262144,

(22) $$\begin{array}{c} secImgY=\text{reshape}\left(\text{pix}Y,512,512\right), \end{array}$$

(23) $$\begin{array}{c} secImg=\text{xor}\left(secImgX,secImgY\right). \end{array}$$

The parameters for the Arnold cat map that perform permutations on the plain image and secret images are the set of *P* and *Q*. Each pair of *P* and *Q* is a modified value of a point on the Henon sequence. These coordinates are real numbers. They converted to integer numbers using multiplied, modular, and absolute operations as described in (8) and (9). By setting quantities *δ* = 12345 and *ϑ* = 67890, *P* and *Q* are in the form of

(24) Pi=abs(⌊x100+i×1014⌋)mod⁡12345,i=1,…,p(q+r),Qi=abs(⌊y100+i×1014⌋)mod⁡67890,i=1,…,p(q+r).

### 4.2. Running Time

Pixel permutation, bit permutation, and pixel modification are three key functions in this cryptosystem. The encryption time depends on the run time of each function and the number of rounds.  Table 2 presents the average run time for one round of each function in this cryptosystem on a 512 × 512 image. Additional tests show a linear relation between running time and number of pixels. The results for 256 × 256 were almost a quarter of the given intervals in Table 2. The total encryption time is calculated by (25) and the decryption time is calculated by (26).  Table 3 is the encryption time of proposed cryptosystem in comparison with recent similar works for a 256 × 256 gray image. Consider

(25) $$\begin{array}{c} T_{E}=\;p*\left(r*T_{BP}+q*T_{PP}+T_{PM}\right), \end{array}$$

(26) TD=p∗q∗TPP+TIPM+(p−1) ×(r∗TIBP+q∗TIPP+TIPM).

### 4.3. Encryption and Decryption Illustrations and Histogram

**Figure 5:**
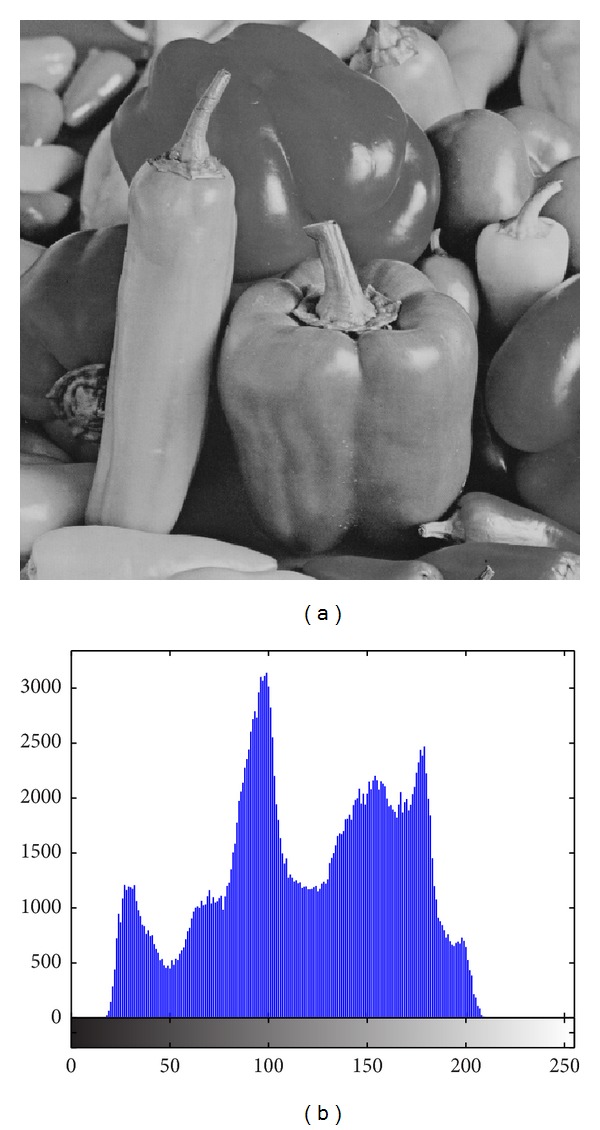
(a) Plain image and (b) its histogram.

**Figure 6:**
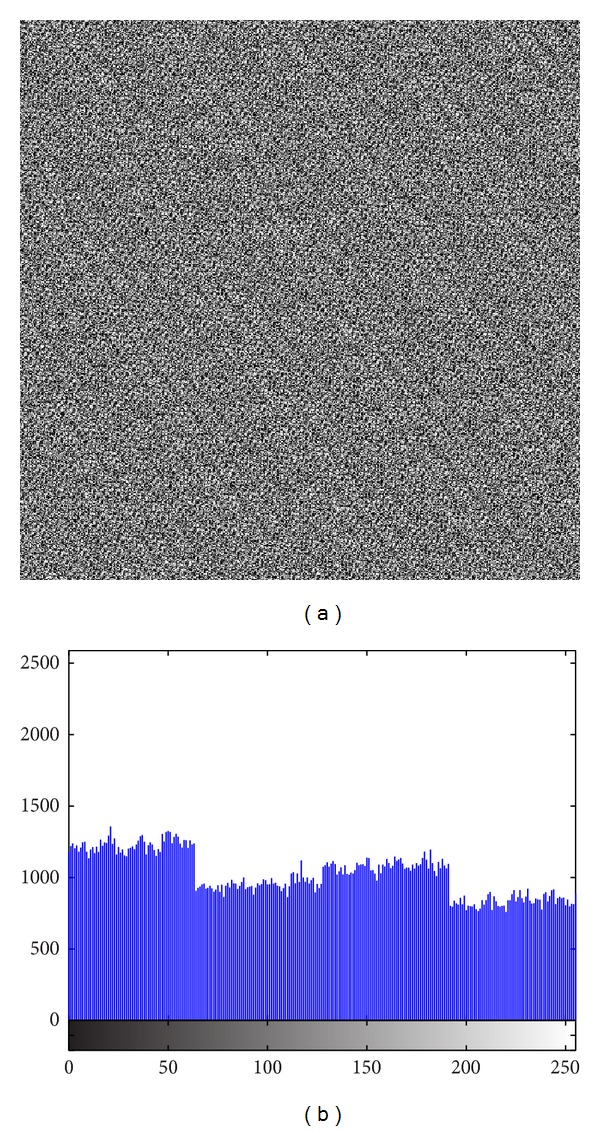
(a) Plain image after four rounds of bit permutation based on Zhu's algorithm and (b) its histogram.

**Figure 7:**
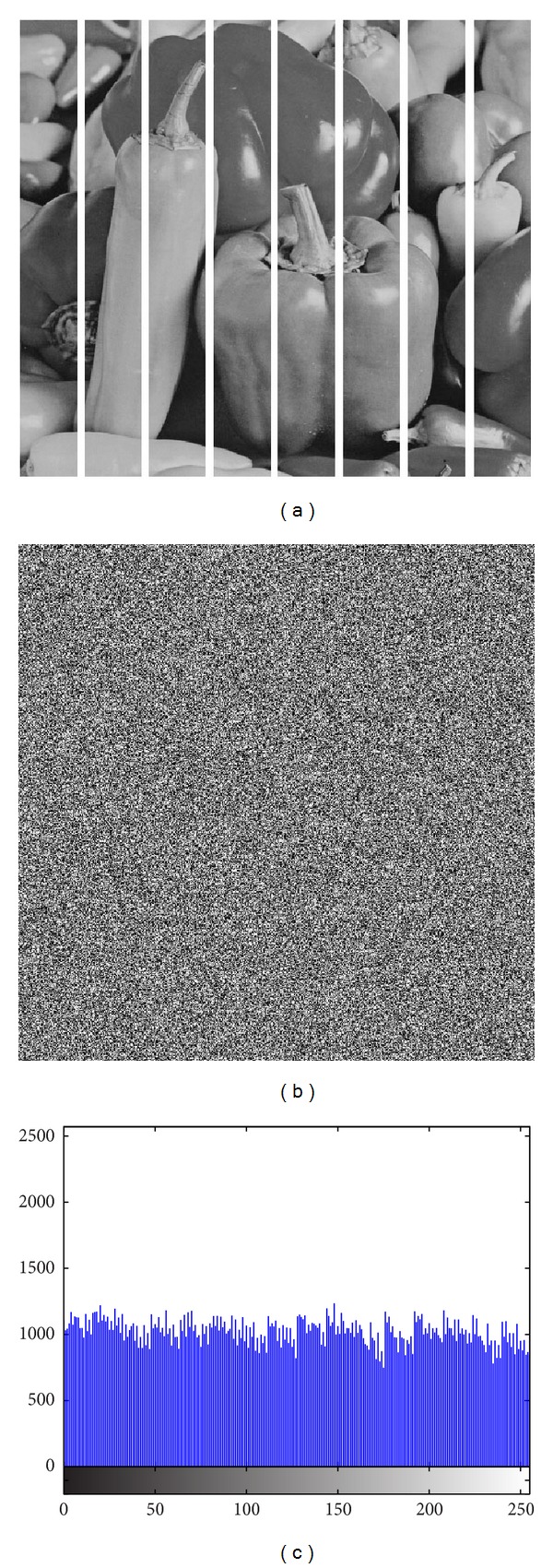
(a) Plain subimages, (b) plain image after four rounds of proposed bit permutation and (c) its histogram.

**Figure 8:**
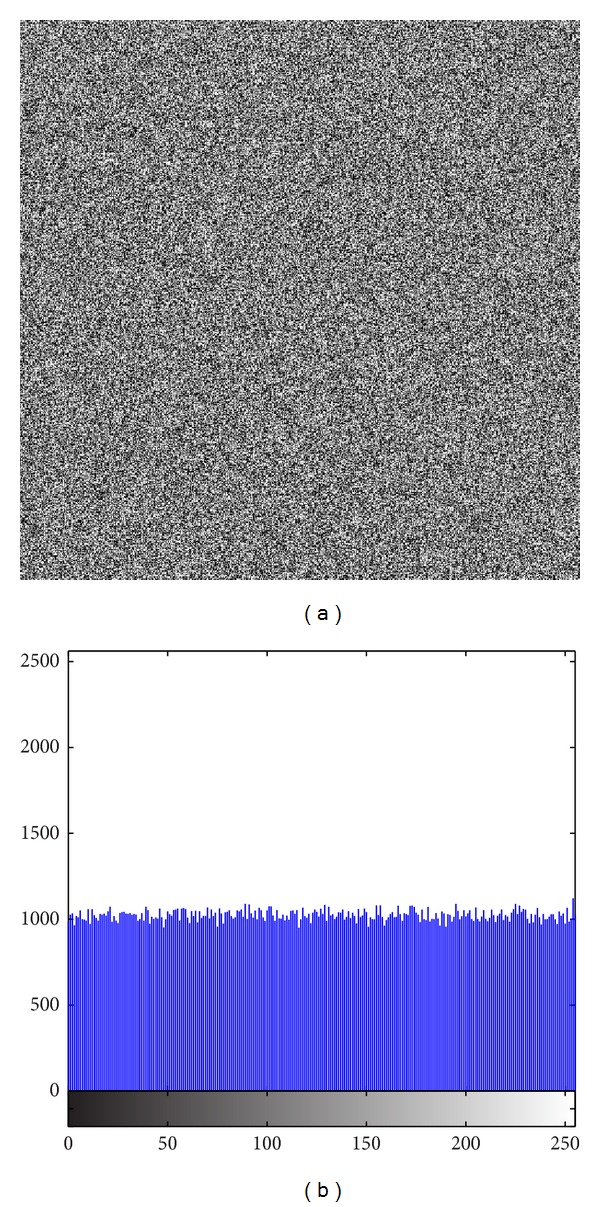
(a) Encrypted image and (b) its histogram.

**Figure 9:**
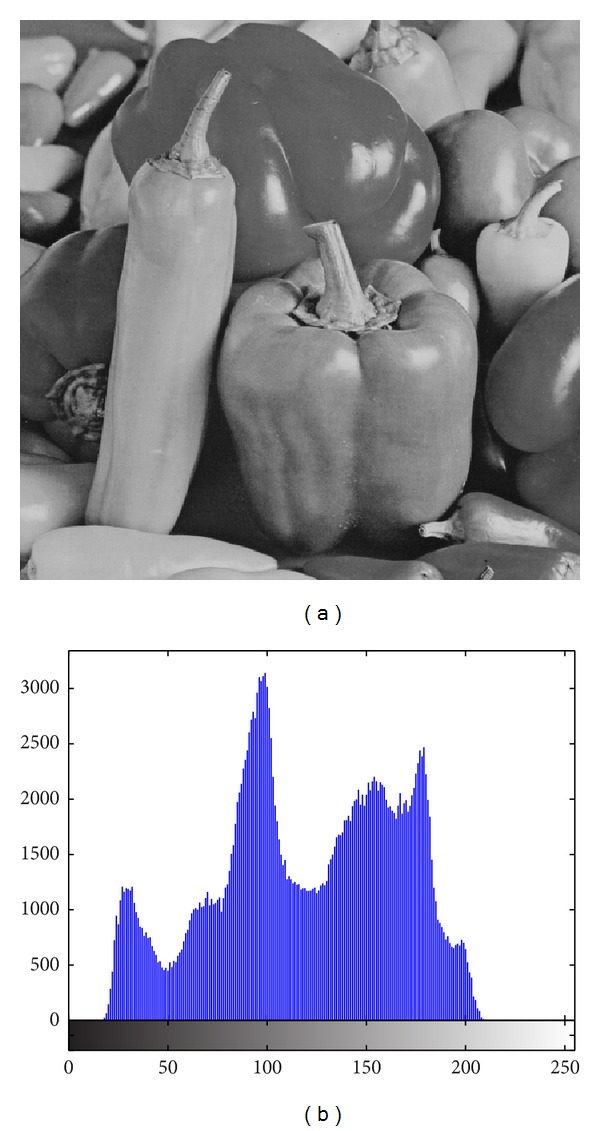
(a) Decrypted image and (b) its histogram.

The image histogram is the graphical illustration of the pixel distribution at different gray levels. A great deal of statistical information regarding the image is extractable from its histogram [45]. The histogram of an encrypted image should have a uniform distribution and be completely different from that of the plain image. This prevents the leakage of any meaningful information from the plain image. In addition to the histogram, the encrypted image must be absolutely unique in appearance without any similar pattern to the original image. Basically, the histogram analysis results are demonstrated for a bit-permuted plain image based on the proposed technique to compare with the proposed scheme by Zhu et al. [9]. Despite the pixel value alteration by Zhu's model, the histogram of the permuted image is not uniform. This is due to the permutation of a group of bits in the same position. According to Shannon's theory, the 8th bit (MSB) pixel values carry almost 50% of the total information of the image. Permuting all of the bits at the same location will not vastly change the image pixels' value. Hence, the plain image, which is shown in Figure 5, is bit permuted for four rounds, and the result and its histogram are illustrated in Figure 6. It seems that there are patterns that still appear in the image. To overcome this vulnerability, a bit permutation scheme is proposed and shuffles the bits entirely and independently of its position.  Figure 7 shows the subimages of the plain image that would be permuted independently. After four rounds of permuting each subimage with altered parameters, the result and its histogram are shown in Figure 7. Visual comparison of images and histograms in Figures 6 and 7 demonstrates the efficacy of the proposed bit-permutation model. Figures 8 and 9 are the images and the histograms after the encryption and decryption process, respectively. The decrypted image is the same as the original one and this technique is found to be lossless.

### 4.4. Key Analysis

#### 4.4.1. Key Space Analysis

The total number of possible keys that an attacker must try to break a cryptosystem is called key space and it should be large enough to prevent brute-force attack. In the proposed cryptosystem, the initial point (*x*_0_, *y*_0_) of the Henon map was used as one of the secret keys. Other control parameters are *γ*,  *λ*, *δ*,   *ϑ*, *p*, *q*, and *r*. These parameters should be kept secret and be used as secret keys.  Table 4 is the upper bound for each variable. A combination of these parameters will provide a large key space of approximately 2^300^ that is sufficient to make brute-force attack infeasible and very large rather than similar works that are compared in Table 5.

#### 4.4.2. Key Sensitivity Analysis

In addition to a sufficiently large key space to protect an encrypted image from brute-force attacks, a strength algorithm should also be absolutely sensitive to both encryption and decryption keys. Changing even one bit in a secret key will cause a completely different result in either the encrypted image or the decrypted image. Key sensitivity is analyzed in both the encryption and the decryption phase. In the encryption phase, the cipher image that results from changing even one bit in any one of the initial values is compared with the encrypted image that resulted before changing the key. The results are given in Table 6. Several experiments were performed and, in each experiment, only one parameter was manipulated while others were unchanged. The changed values and the difference rates for the produced images are listed in the table.

In the decryption phase, key sensitivity means that the encrypted image cannot be decrypted by slight variations in the secret key. Based on the results in Table 7, changing even one bit in the decryption key will result in a wholly different decrypted image.

### 4.5. Statistical Analysis

Statistical analysis can extract the relationships between the original and the encrypted image. Shannon in his theory of information and communication [45] proved that it is possible to break many types of cryptograms by statistical analysis. This can be thwarted by dissipating the redundancy in the structure of the message by diffusion or by increasing the complexity of the relationship between the encrypted message and the secret key by confusion. Either confusion or diffusion is presented in the proposed cryptosystem to frustrate statistical attacks.

#### 4.5.1. Correlation Analysis

**Figure 10:**
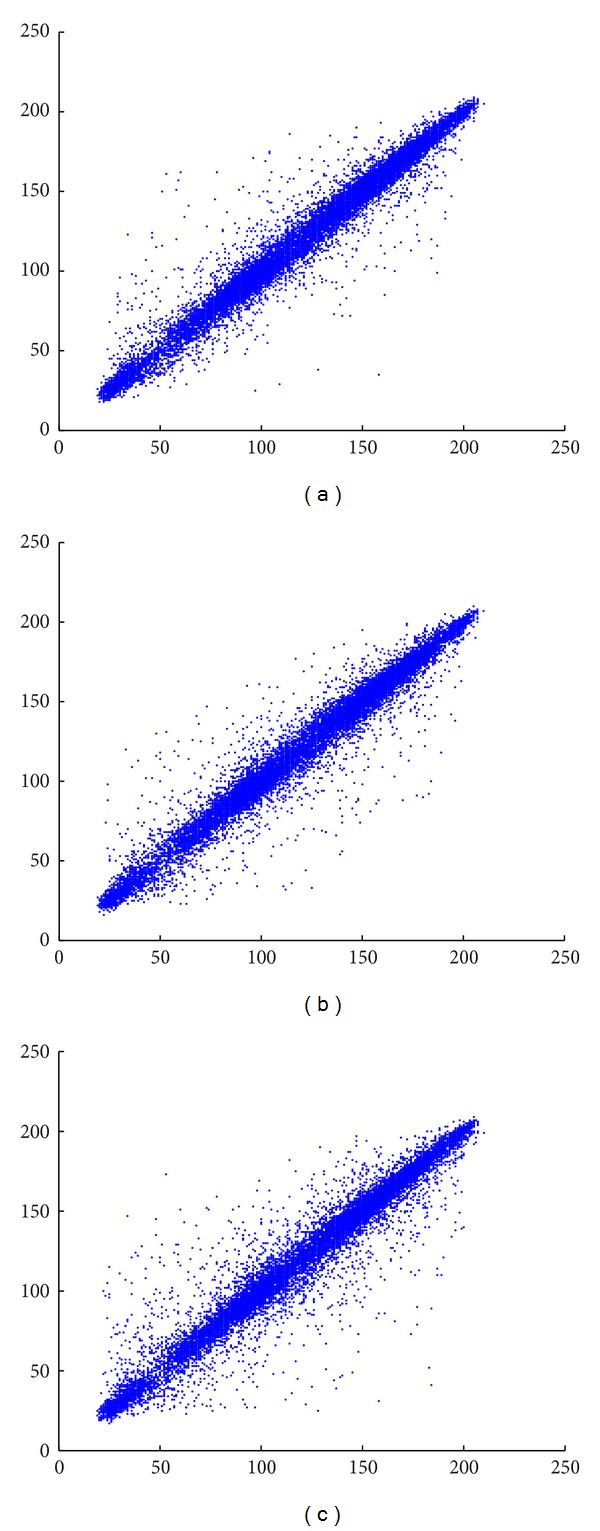
Correlation of plain image's pixels in (a) horizontal, (b) vertical, and (c) diagonal position.

**Figure 11:**
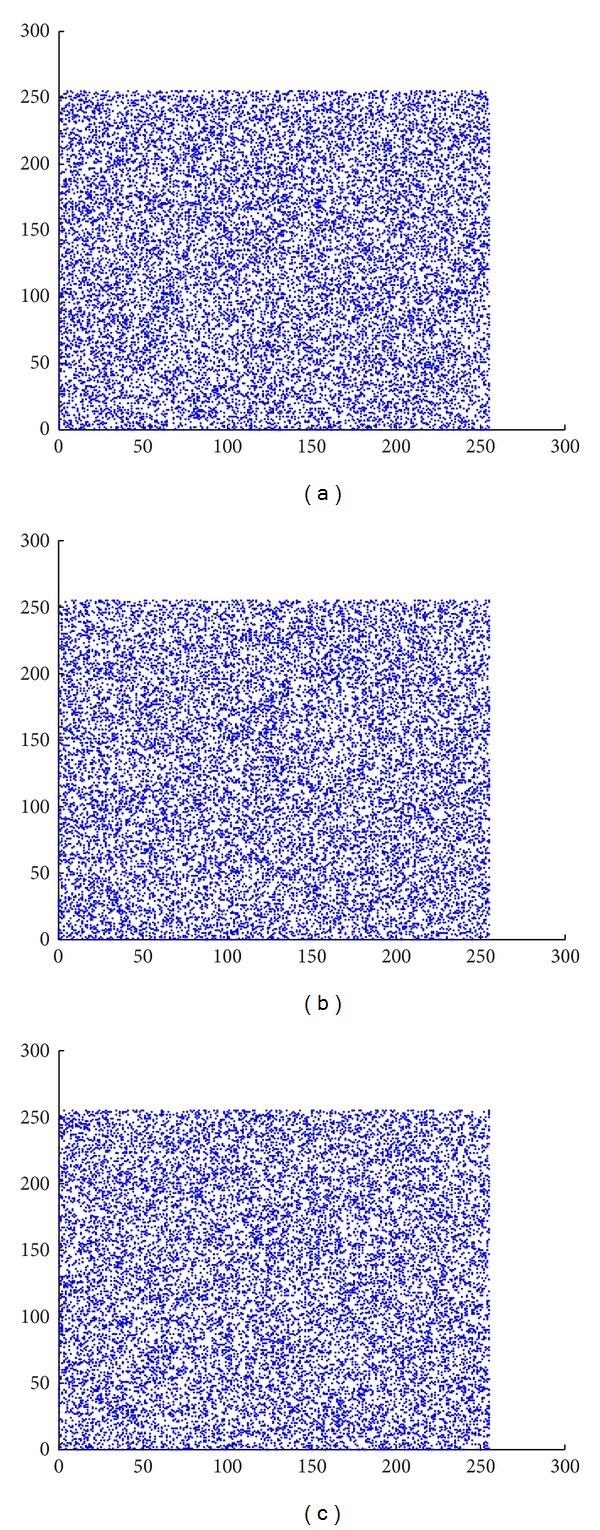
Correlation of cipher image's pixels in (a) horizontal, (b) vertical, and (c) diagonal position.

Two adjoining pixels in a regular image are strongly correlated in horizontal, vertical, and diagonal positions. Scatter plots in Figures 10 and 11 reveal the correlation of two adjacent pixels in horizontal, vertical, and diagonal distributions in the plain and the cipher image, respectively. Correlation coefficients are calculated for test images by (27) and the results for plain images and cipher images are listed in Table 14. For an ordinary image, the correlation coefficients are very close to 1, which is the highest possible value. The produced encrypted image is ideal and resists statistical attack if the correlation coefficients are very low and close to 0. Consider

(27) $$\begin{array}{c} r_{x,y}=\frac{\sum \left(x_{i}-\overset{-}{x}\right)\left(y_{i}-\overset{-}{y}\right)}{\sqrt{\sum {\left(x_{i}-\overset{-}{x}\right)}^{2}\sum {\left(y_{i}-\overset{-}{y}\right)}^{2}}}. \end{array}$$

#### 4.5.2. Entropy Analysis

Entropy is a statistical parameter that is defined to measure the uncertainly and randomness of a bundle of data. According to Shannon theory, image entropy is the number of bits that is necessary to encode every pixel of the image. The optimal value for entropy of an encrypted image is ~8. This quantity describes the random pattern and texture of pixels in an encrypted image and is calculated by

(28) $$\begin{array}{c} \text{entropy}=\;\overset{n}{\underset{i=0}{\sum }}P_{i}\;\log_{2}P_{i}, \end{array}$$

where *n* is the total number of gray levels (i.e., 256) and *P*_*i*_ is the probability of incidence of intensity *i* in the current image. *P*_*i*_ is the number of pixels with intensity *i* divided by the total number of pixels. The base-2 logarithm will present the calculated entropy in bits. The entropy values for plain images and encrypted images are given in Table 14.

### 4.6. Differential Analysis

For the purpose of differential attack, an attacker changes a specific pixel in the plain image and traces the differences in the analogous encrypted image to find a meaningful relation. This is also known as a chosen-plaintext attack. A robust encrypted image must be sensitive to minor changes and even changing one bit in the plain image should result in a wide range of changes in the cipher image.

The NPCR measures the number of pixels change rate in an encrypted image when 1 bit is changed in the plain image. This parameter is calculated by (29) and for an ideal encryption algorithm it is 1. Consider

(29) NPCR=1m×n∑i=1m∑j=1nf(i,j)  ×  100,f(i,j)={0,if  c1(i,j)=c2(i,j),1,if  c1(i,j)≠c2(i,j),

where *c*_1_ and *c*_2_ are obtained by encrypting two *m* × *n* plain images and one random bit dissimilarity.

The UACI in differential analysis is the unified average changing intensity between two encrypted images with a difference in only one bit in corresponding plain images. The UACI can be calculated by (30):

(30) $$\begin{array}{c} \text{UACI}=\frac{1}{m\times n}\;\overset{m}{\underset{i=1}{\sum }}\overset{n}{\underset{j=1}{\sum }}\frac{\left|c_{1}\left(i,j\right)-c_{2}\left(i,j\right)\right|}{255}\;\times 100. \end{array}$$

To evaluate the sensitivity of the proposed algorithm to differential attacks, a random bit is changed in the plain image. Encrypting two plain images with a difference in only one bit produces two encrypted images. The rates of pixel and intensity differences in the two encrypted images are calculated. Tables 8, 9, and 10 present calculated UACI and NPCR values for different combinations of *p*, *q*, and *r* to trade off the encryption speed and the overall rounds to find a threshold that achieves the highest rate. The following tables are related to the Peppers image. From Tables 8 and 9, it was concluded that increasing the value of the parameter *q* that is related to the pixel permutation rounds does not affect the NPCR and UACI values. These values depend only on *p* and *r*. However, to increase the level of confusion and increase the key space, pixel permutation is required. The results in Table 10 were used to find the minimum values for *p* and *r* that result in the ideal value for NPCR and UACI with the smallest run-time. It was concluded that at least three rounds of *p* were required to obtain the highest values for UACI and NPCR. After the 3rd row, all of the combinations are ideal. Different combinations of *p*, *q*, and *r* are calculated with (25) and the combination of (*p*, *q*, *r*) that encrypts the image with the smallest run-time is (3, 1, 1).

**Figure 12:**
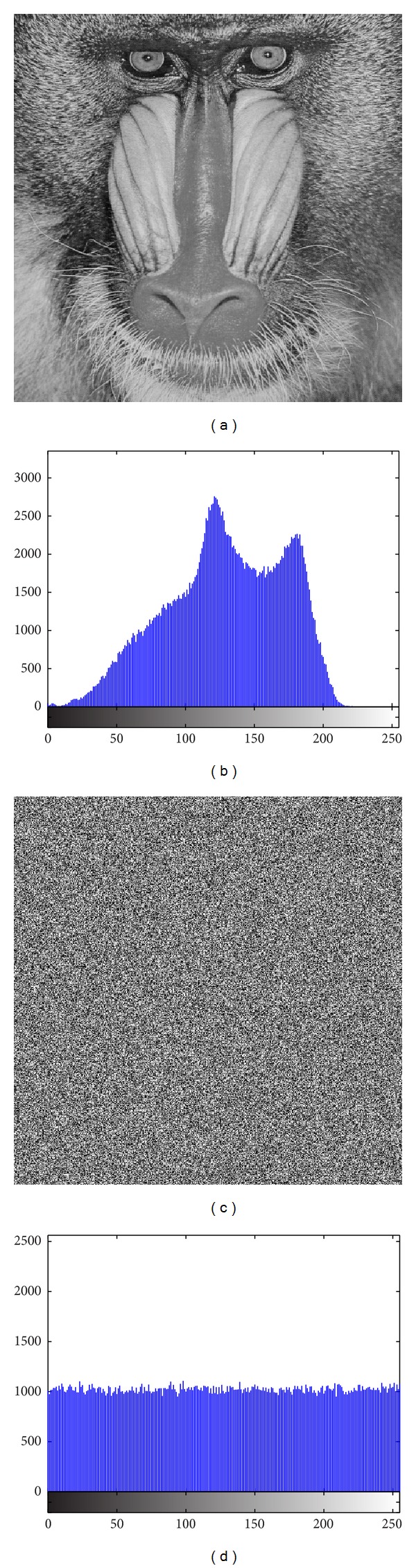
(a) Baboon image and (b) its histograms and (c) encrypted image and (d) its histogram.

**Figure 13:**
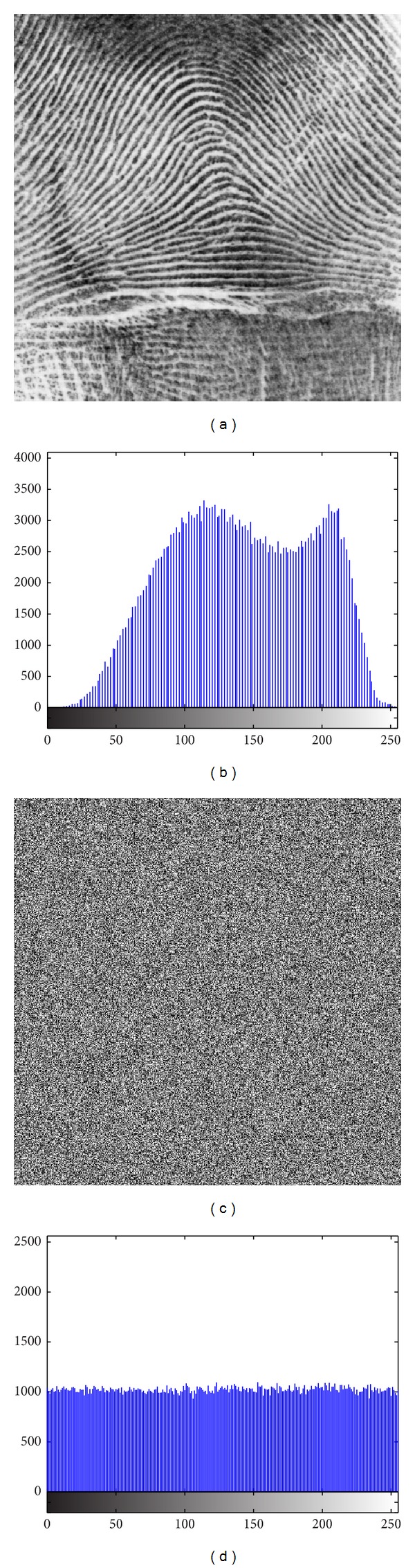
(a) Fingerprint image and (b) its histograms and (c) encrypted image and (d) its histogram.

**Figure 14:**
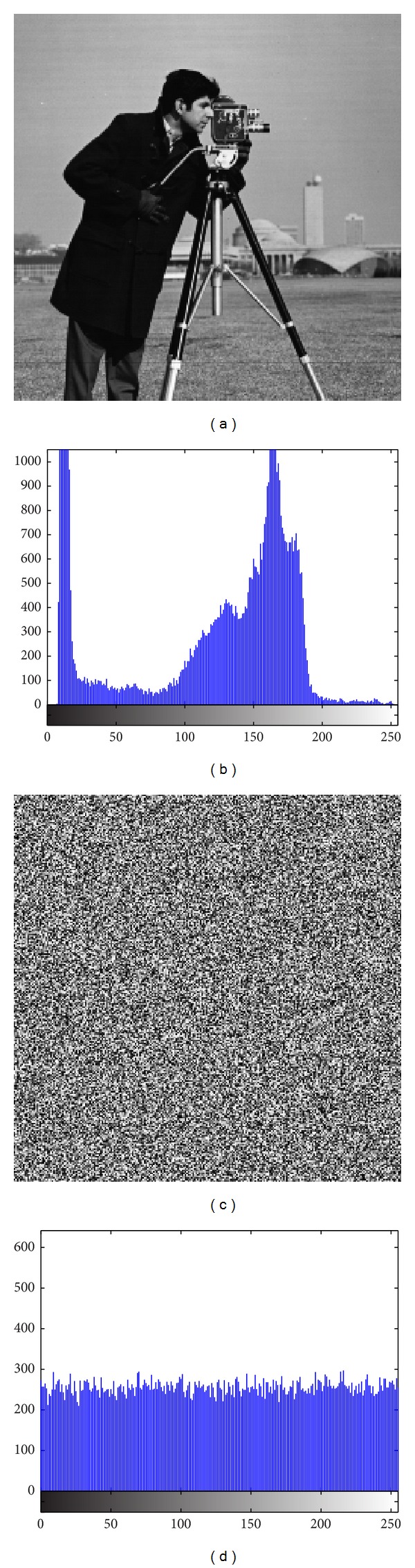
(a) Cameraman image and (b) its histograms and (c) encrypted image and (d) its histogram.

**Figure 15:**
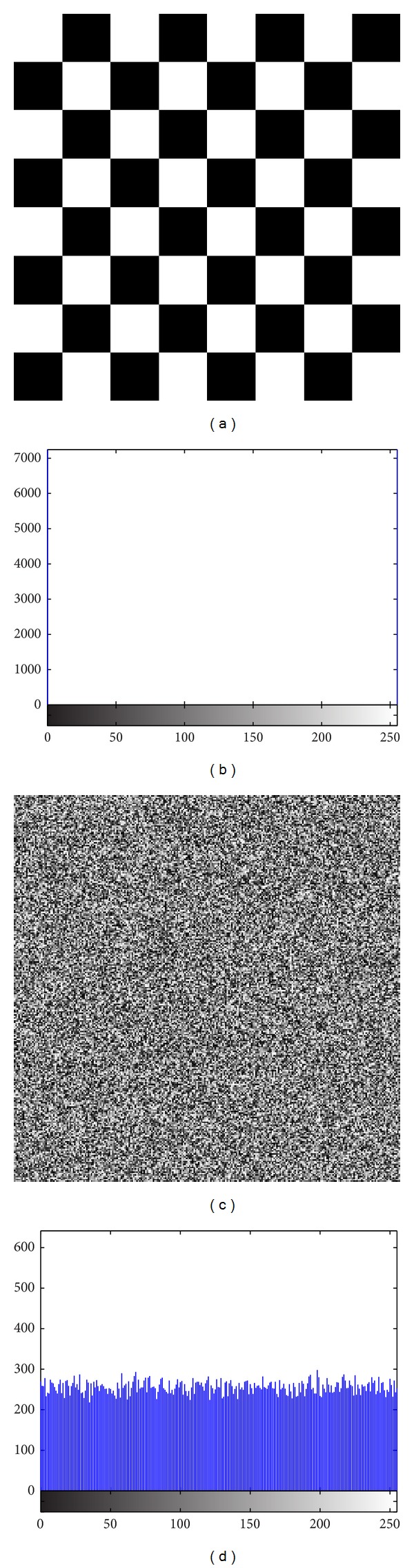
(a) Chess-plate image and (b) its histograms and (c) encrypted image and (d) its histogram.

In addition to Peppers, the same experiments were performed on Baboon, Figure 12, Fingerprint, Figure 13, Cameraman, Figure 14, and Chess-plate, Figure 15. The results of the experiments on these three images are investigated to determine the strength of the proposed cryptosystem. Tables 11, 12, 13, and 14 list the calculated values of UACI and NPCR for different combinations of *p* and *r* in Baboon, Fingerprint, Cameraman, and Chess-plate images, respectively. Because the results were similar to other combinations of the Pepper image, the other similar tables were discarded and only the set of *p* and *r* was surveyed. The results for entropy, correlation, and brief of the UACI and NPCR are listed in Table 15.

## 5. Conclusion and Future Works

In this paper, a new chaos-based cryptosystem has been proposed for encrypting images. The Arnold cat map and the Henon map are two discrete chaotic maps that are used in this scheme. Bit shuffling and pixel shuffling are reversible transformations that are performed using the Arnold cat map with various secret parameters. Improving the randomness of transformation and the efficiency of bit permutation are two advantages of this cryptosystem that increases the strength of the ciphered image in comparison with previous works. Iterating the Arnold cat map with different parameters at each round prevents undesirable reconstruction of the input image. These parameters are generated by the Henon map with secret initial values. The points generated by the Henon map are also applied to create secret images for more confusion and diffusion and to increase the key space. Sequential XOR of the bit-permuted plain image and the pixel-permuted secret image is another phase of modifying the pixels values. This creates a slight distortion in the plain image to prevent successful differential attacks. The results of security analysis of five images demonstrate the resistance of the encrypted image to statistical attacks and to the chosen-plaintext attack. In addition, a sufficiently large key space makes a brute force attack impractical. As the future work, the proposed cryptosystem in this paper will combine with a public key technique such as ECC or RSA to propose a hybrid encryption method. This technique is a chaotic asymmetric cryptosystem.

## Figures and Tables

**Table 1:** Applied chaos maps in some proposed image encryption techniques.

	ACM	Logistic	Henon	Lorenz	Baker	Chen	Tent	CML	Standard map
Zhu et al. [9]	×	×							
Xu et al. [10]						×			
Zhang and Cao [11]	×			×					
Fu et al. [12]	×								
Zhang et al. [13]	×	×	×						
Ghebleh et al. [14]	×						×		
Elshamy et al. [15]					×				
Ye and Zhou [16]							×	×	
Ye and Zhou [17]							×	×	
Wang et al. [18]					×				
Al-Maadeed et al. [19]		×							
Patidar et al. [20]		×							×
Wong et al. [21]									×
Guanghuia et al. [22]		×							
Zhang et al. [23]		×							
Liu et al. [24]	×	×							

**Table 2:** Evaluated running time for main functions in proposed cryptosystem.

Variable	Function	Running time for one round (ms)
*T*_BP_	Bit permutation running time	62
*T*_PP_	Pixel permutation running time	13
*T*_PM_	Pixel modification running time	4
*T*_IBP_	Inverse bit permutation running time	75
*T*_IPP_	Inverse pixel permutation running time	23
*T*_IPM_	Inverse pixel modification running time	4

**Table 3:** Comparison of encryption time for proposed algorithm with recent similar works.

	Proposed scheme	[12]	[10]	[25]	[26]	[27]
Encryption time (ms)	19.75	23	22	20.79	32	52

**Table 4:** Secret parameters length in bit.

Parameter	Length (bit)
*p*	10
*q*	10
*r*	10
*γ*	48
*λ*	48
*δ*	24
*ϑ*	24
*x*_0_	64
*y*_0_	64

**Table 5:** Comparison of encryption time for proposed algorithm with recent similar works.

	Proposed scheme	[12]	[10]	[25]	[26]	[28]	[27]
Key space	2^300^	2^153^	2^120^	2^128^	2^186^	2^199^	2^256^

**Table 6:** Difference rates of two encrypted images with slight change in a parameter.

Parameter	Initial value	Changed value	Encrypted images difference rate
*p*	6	7	99.59%
*q*	2	1	99.60%
*r*	2	1	99.62%
Γ	12345678	12345679	99.58%
*λ*	87654321	87654320	99.62%
*δ*	12345	12346	99.63%
*ϑ*	67890	67891	99.60%
*x*_0_	1.21000001	1.21	99.59%
*y*_0_	0.36000001	0.36	99.60%

**Table 7:** Difference rate of two decrypted images with slight change in a parameter.

Parameter	Encryption parameters	Decryption parameters	Decrypted images difference rate
p	6	5	99.59%
q	2	1	99.62%
r	2	1	99.59%
γ	12345678	12345677	99.62%
λ	87654321	87654322	99.58%
δ	12345	12344	99.59%
ϑ	67890	67889	99.60%
x_0_	1.21000001	1.21000002	99.60%
y_0_	0.36000001	0.36000002	99.62%

**Table 8:** Calculated UACI and NPCR for different combinations of *q* and *r* while *p* = 1 in Peppers.

*q*/*r*		1	2	3	4	5	6	7	8	9	10
1	UACI	5.1163	2.7810	2.0331	1.1860	1.5810	6.1305	1.4349	3.7728	3.3215	0.0564
	NPCR	81.5414	44.3218	32.4032	18.9026	25.1965	97.7051	22.8695	60.1284	52.9366	0.8984
2	UACI	5.1163	2.7810	2.0331	1.1860	1.5810	6.1305	1.4349	3.7728	3.3215	0.0564
	NPCR	81.5414	44.3218	32.4032	18.9026	25.1965	97.7051	22.8695	60.1284	52.9366	0.8984
3	UACI	5.1163	2.7810	2.0331	1.1860	1.5810	6.1305	1.4349	3.7728	3.3215	0.0564
	NPCR	81.5414	44.3218	32.4032	18.9026	25.1965	97.7051	22.8695	60.1284	52.9366	0.8984
4	UACI	5.1163	2.7810	2.0331	1.1860	1.5810	6.1305	1.4349	3.7728	3.3215	0.0564
	NPCR	81.5414	44.3218	32.4032	18.9026	25.1965	97.7051	22.8695	60.1284	52.9366	0.8984
5	UACI	5.1163	2.7810	2.0331	1.1860	1.5810	6.1305	1.4349	3.7728	3.3215	0.0564
	NPCR	81.5414	44.3218	32.4032	18.9026	25.1965	97.7051	22.8695	60.1284	52.9366	0.8984
6	UACI	5.1163	2.7810	2.0331	1.1860	1.5810	6.1305	1.4349	3.7728	3.3215	0.0564
	NPCR	81.5414	44.3218	32.4032	18.9026	25.1965	97.7051	22.8695	60.1284	52.9366	0.8984
7	UACI	5.1163	2.7810	2.0331	1.1860	1.5810	6.1305	1.4349	3.7728	3.3215	0.0564
	NPCR	81.5414	44.3218	32.4032	18.9026	25.1965	97.7051	22.8695	60.1284	52.9366	0.8984
8	UACI	5.1163	2.7810	2.0331	1.1860	1.5810	6.1305	1.4349	3.7728	3.3215	0.0564
	NPCR	81.5414	44.3218	32.4032	18.9026	25.1965	97.7051	22.8695	60.1284	52.9366	0.8984
9	UACI	5.1163	2.7810	2.0331	1.1860	1.5810	6.1305	1.4349	3.7728	3.3215	0.0564
	NPCR	81.5414	44.3218	32.4032	18.9026	25.1965	97.7051	22.8695	60.1284	52.9366	0.8984
10	UACI	5.1163	2.7810	2.0331	1.1860	1.5810	6.1305	1.4349	3.7728	3.3215	0.0564
	NPCR	81.5414	44.3218	32.4032	18.9026	25.1965	97.7051	22.8695	60.1284	52.9366	0.8984

**Table 9:** Calculated UACI and NPCR for different combinations of *q* and *p* while *r* = 1 in Peppers.

*q*/*p*		1	2	3	4	5	6	7	8	9	10
1	UACI	5.1163	16.2147	**33.4573**	**33.4727**	**33.4075**	**33.4355**	**33.4484**	**33.5372**	**33.4775**	**33.4735**
	NPCR	81.5414	89.1617	**99.6342**	**99.6208**	**99.6059**	**99.6231**	**99.5930**	**99.6277**	**99.5930**	**99.6174**
2	UACI	5.1163	16.2173	**33.4317**	**33.4902**	**33.4462**	**33.4644**	**33.5382**	**33.4616**	**33.5023**	**33.4503**
	NPCR	81.5414	89.1617	**99.6342**	**99.6208**	**99.6059**	**99.6231**	**99.5930**	**99.6277**	**99.5930**	**99.6174**
3	UACI	5.1163	16.2191	**33.3994**	**33.5234**	**33.4093**	**33.4373**	**33.4497**	**33.4928**	**33.5363**	**33.5198**
	NPCR	81.5414	89.1617	**99.6342**	**99.6208**	**99.6059**	**99.6231**	**99.5930**	**99.6277**	**99.5930**	**99.6174**
4	UACI	5.1163	16.2003	**33.4263**	**33.4481**	**33.3943**	**33.4181**	**33.5062**	**33.4770**	**33.4784**	**33.5147**
	NPCR	81.5414	89.1617	**99.6342**	**99.6208**	**99.6059**	**99.6231**	**99.5930**	**99.6277**	**99.5930**	**99.6174**
5	UACI	5.1163	16.1772	**33.4520**	**33.4995**	**33.3855**	**33.4539**	**33.4610**	**33.5291**	**33.4940**	**33.4577**
	NPCR	81.5414	89.1617	**99.6342**	**99.6208**	**99.6059**	**99.6231**	**99.5930**	**99.6277**	**99.5930**	**99.6174**
6	UACI	5.1163	16.1845	**33.4262**	**33.5164**	**33.3721**	**33.4232**	**33.4606**	**33.5161**	**33.4626**	**33.4400**
	NPCR	81.5414	89.1617	**99.6342**	**99.6208**	**99.6059**	**99.6231**	**99.5930**	**99.6277**	**99.5930**	**99.6174**
7	UACI	5.1163	16.1885	**33.4141**	**33.4955**	**33.4241**	**33.4615**	**33.4464**	**33.5113**	**33.4486**	**33.4434**
	NPCR	81.5414	89.1617	**99.6342**	**99.6208**	**99.6059**	**99.6231**	**99.5930**	**99.6277**	**99.5930**	**99.6174**
8	UACI	5.1163	16.1882	**33.4694**	**33.4696**	**33.3941**	**33.4834**	**33.5019**	**33.4556**	**33.4589**	**33.5000**
	NPCR	81.5414	89.1617	**99.6342**	**99.6208**	**99.6059**	**99.6231**	**99.5930**	**99.6277**	**99.5930**	**99.6174**
9	UACI	5.1163	16.1954	**33.4277**	**33.4527**	**33.3872**	**33.5124**	**33.4510**	**33.4750**	**33.4249**	**33.5083**
	NPCR	81.5414	89.1617	**99.6342**	**99.6208**	**99.6059**	**99.6231**	**99.5930**	**99.6277**	**99.5930**	**99.6174**
10	UACI	5.1163	16.2147	**33.4573**	**33.4727**	**33.4075**	**33.4355**	**33.4484**	**33.5372**	**33.4775**	**33.4735**
	NPCR	81.5414	89.1617	**99.6342**	**99.6208**	**99.6059**	**99.6231**	**99.5930**	**99.6277**	**99.5930**	**99.6174**

**Table 10:** Calculated UACI and NPCR for different combinations of *p* and *r* while *q* = 1 in Peppers.

*p*/*r*		1	2	3	4	5	6	7	8	9	10
1	UACI	5.1163	2.7810	2.0331	1.1860	1.5810	6.1305	1.4349	3.7728	3.3215	0.0564
	NPCR	81.5414	44.3218	32.4032	18.9026	25.1965	97.7051	22.8695	60.1284	52.9366	0.8984
2	UACI	16.2119	2.0258	0.9736	0.4904	0.8663	32.6779	0.4922	3.8314	4.1271	0.1998
	NPCR	89.1617	85.5148	81.7390	75.0000	76.3233	94.8944	75.3403	86.6261	89.8193	50.9499
3	UACI	**33.3879**	**32.8273**	**34.7519**	**32.7804**	**33.5263**	**33.5459**	**33.3332**	**34.4429**	**33.5705**	**33.2778**
	NPCR	**99.6342**	**99.5850**	**99.6552**	**99.5987**	**99.6357**	**99.6017**	**99.6098**	**99.6044**	**99.6033**	**99.5075**
4	UACI	**33.4882**	**33.3894**	**33.4144**	**33.4373**	**33.5037**	**33.5170**	**33.4163**	**33.4185**	**33.4494**	**33.5380**
	NPCR	**99.6208**	**99.6124**	**99.5979**	**99.5918**	**99.6075**	**99.5892**	**99.6185**	**99.6075**	**99.5960**	**99.5995**
5	UACI	**33.3994**	**33.3844**	**33.5083**	**33.4886**	**33.4846**	**33.5354**	**33.4447**	**33.5059**	**33.3940**	**33.4656**
	NPCR	**99.6059**	**99.6124**	**99.6258**	**99.6159**	**99.6033**	**99.6067**	**99.6101**	**99.6166**	**99.6120**	**99.5953**
6	UACI	**33.4314**	**33.4144**	**33.4623**	**33.3797**	**33.4872**	**33.5663**	**33.4599**	**33.4731**	**33.4311**	**33.4141**
	NPCR	**99.6231**	**99.6296**	**99.6250**	**99.6006**	**99.6208**	**99.6265**	**99.6037**	**99.6040**	**99.5926**	**99.6120**
7	UACI	**33.4744**	**33.4282**	**33.4541**	**33.5446**	**33.4563**	**33.4700**	**33.4543**	**33.4240**	**33.4748**	**33.4333**
	NPCR	**99.5930**	**99.5968**	**99.6128**	**99.6017**	**99.6082**	**99.6021**	**99.6311**	**99.6132**	**99.5964**	**99.6185**
8	UACI	**33.4535**	**33.3790**	**33.5941**	**33.5118**	**33.5437**	**33.4958**	**33.5057**	**33.5276**	**33.4622**	**33.4553**
	NPCR	**99.6277**	**99.5972**	**99.5758**	**99.6044**	**99.6094**	**99.5983**	**99.6170**	**99.6136**	**99.5888**	**99.5983**
9	UACI	**33.4703**	**33.4465**	**33.4612**	**33.5285**	**33.5804**	**33.4783**	**33.4213**	**33.4463**	**33.4063**	**33.4409**
	NPCR	**99.5930**	**99.5975**	**99.6006**	**99.6094**	**99.6185**	**99.6262**	**99.5903**	**99.6052**	**99.6082**	**99.6071**
10	UACI	**33.4631**	**33.4266**	**33.3962**	**33.4113**	**33.4824**	**33.5212**	**33.5256**	**33.4979**	**33.3891**	**33.4482**
	NPCR	**99.6174**	**99.6109**	**99.6178**	**99.6105**	**99.6212**	**99.6071**	**99.5857**	**99.6014**	**99.6071**	**99.5960**

**Table 11:** Calculated UACI and NPCR for different combinations of *p* and *r* while *q* = 1 in Baboon.

*p*/*r*		1	2	3	4	5	6	7	8	9	10
1	UACI	34.5764	18.5622	1.9700	39.7193	43.0302	12.3125	4.4068	40.1488	48.6447	20.7776
	NPCR	68.8828	36.9793	3.9246	79.1283	85.7243	24.5289	8.7791	79.9839	96.9093	41.3929
2	UACI	7.9196	0.9566	0.1960	15.6506	16.3404	0.5035	0.1959	14.2109	32.8500	1.7190
	NPCR	87.5687	81.3938	49.9817	93.7302	93.2835	76.1761	49.9420	86.6169	93.3601	67.2352
3	UACI	**33.4245**	**32.9577**	**32.9665**	**33.6173**	**33.4770**	**33.5504**	**33.4176**	**32.8382**	**33.4229**	**32.5148**
	NPCR	**99.6040**	**99.6105**	**99.6094**	**99.6319**	**99.6090**	**99.6128**	**99.6162**	**99.6017**	**99.5922**	**99.5224**
4	UACI	**33.5433**	**33.4850**	**33.4328**	**33.4770**	**33.4972**	**33.4388**	**33.4096**	**33.4726**	**33.4217**	**33.4147**
	NPCR	**99.6140**	**99.6307**	**99.6166**	**99.6136**	**99.6037**	**99.6071**	**99.6059**	**99.5964**	**99.6056**	**99.6235**
5	UACI	**33.4336**	**33.4809**	**33.4824**	**33.4264**	**33.4568**	**33.5143**	**33.4470**	**33.3774**	**33.4297**	**33.3378**
	NPCR	**99.6151**	**99.5892**	**99.6128**	**99.6189**	**99.6273**	**99.5922**	**99.6407**	**99.6044**	**99.5819**	**99.6120**
6	UACI	**33.4377**	**33.4569**	**33.4803**	**33.4672**	**33.5039**	**33.4647**	**33.4143**	**33.5158**	**33.4548**	**33.4117**
	NPCR	**99.6021**	**99.5987**	**99.6178**	**99.5975**	**99.6174**	**99.6155**	**99.6090**	**99.6067**	**99.6140**	**99.6143**
7	UACI	**33.4281**	**33.4751**	**33.4826**	**33.4711**	**33.4626**	**33.4911**	**33.4976**	**33.4263**	**33.4891**	**33.5239**
	NPCR	**99.6052**	**99.6120**	**99.5922**	**99.5892**	**99.5998**	**99.6334**	**99.6040**	**99.6052**	**99.6258**	**99.6120**
8	UACI	**33.4534**	**33.5000**	**33.4770**	**33.4986**	**33.4616**	**33.4514**	**33.4376**	**33.4205**	**33.4672**	**33.5179**
	NPCR	**99.6265**	**99.5892**	**99.5953**	**99.6082**	**99.6101**	**99.6059**	**99.6014**	**99.6334**	**99.6258**	**99.6128**
9	UACI	**33.4524**	**33.5607**	**33.4740**	**33.5257**	**33.3783**	**33.5049**	**33.4948**	**33.4504**	**33.4197**	**33.4726**
	NPCR	**99.6223**	**99.6204**	**99.6002**	**99.5934**	**99.6124**	**99.5991**	**99.6025**	**99.5911**	**99.5953**	**99.6094**
10	UACI	**33.3259**	**33.4977**	**33.3791**	**33.5318**	**33.5857**	**33.4664**	**33.4249**	**33.4084**	**33.5136**	**33.5256**
	NPCR	**99.6155**	**99.6353**	**99.6536**	**99.6338**	**99.5850**	**99.5987**	**99.6536**	**99.6063**	**99.6155**	**99.6353**

**Table 12:** Calculated UACI and NPCR for different combinations of *p* and *r* while *q* = 1 in Fingerprint.

*p*/*r*		1	2	3	4	5	6	7	8	9	10
1	UACI	0.1102	0.2833	0.2429	0.3697	0.3291	0.4672	0.6168	0.1744	0.0714	0.1102
	NPCR	14.0533	36.1149	30.9715	47.1390	41.9651	59.5711	78.6449	22.2366	9.0992	14.0533
2	UACI	0.4539	1.0997	1.0502	2.0179	2.0101	4.0957	16.3335	0.4905	0.1923	0.4539
	NPCR	71.9505	85.4733	86.7638	85.9703	84.3555	88.2622	89.8018	75.0031	49.0311	71.9505
3	UACI	**32.7711**	**33.2829**	**23.0805**	**33.4200**	**33.3053**	**33.4104**	**33.9360**	**33.4891**	**34.1633**	**32.7711**
	NPCR	**99.6140**	**99.6277**	**99.3843**	**99.6178**	**99.5754**	**99.6014**	**99.6368**	**99.6098**	**99.7284**	**99.6140**
4	UACI	**33.5087**	**33.4496**	**33.4820**	**33.5291**	**33.3559**	**33.4629**	**33.5104**	**33.4655**	**33.4892**	**33.5087**
	NPCR	**99.6105**	**99.6220**	**99.6216**	**99.6178**	**99.6067**	**99.6181**	**99.6120**	**99.5991**	**99.6105**	**99.6105**
5	UACI	**33.5129**	**33.4573**	**33.4886**	**33.4295**	**33.5528**	**33.5534**	**33.4449**	**33.4749**	**33.5176**	**33.5129**
	NPCR	**99.6117**	**99.6181**	**99.5953**	**99.6269**	**99.5880**	**99.6021**	**99.6094**	**99.6166**	**99.5991**	**99.6117**
6	UACI	**33.4461**	**33.4562**	**33.5025**	**33.5266**	**33.4495**	**33.4262**	**33.4276**	**33.4117**	**33.4122**	**33.4461**
	NPCR	**99.5983**	**99.6094**	**99.6044**	**99.5956**	**99.5930**	**99.6132**	**99.6391**	**99.6204**	**99.5926**	**99.5983**
7	UACI	**33.3924**	**33.5282**	**33.4733**	**33.4923**	**33.5048**	**33.4279**	**33.4765**	**33.5338**	**33.4224**	**33.3924**
	NPCR	**99.6265**	**99.6075**	**99.6014**	**99.6273**	**99.6212**	**99.6075**	**99.5922**	**99.6101**	**99.6155**	**99.6265**
8	UACI	**33.4546**	**33.4874**	**33.5073**	**33.4015**	**33.4098**	**33.4831**	**33.3897**	**33.4127**	**33.5300**	**33.4546**
	NPCR	**99.6063**	**99.6025**	**99.6132**	**99.6159**	**99.6147**	**99.6292**	**99.5827**	**99.6315**	**99.5949**	**99.6063**
9	UACI	**33.5202**	**33.4528**	**33.5220**	**33.4781**	**33.4966**	**33.4986**	**33.4642**	**33.4896**	**33.5301**	**33.5202**
	NPCR	**99.6223**	**99.6201**	**99.5995**	**99.6304**	**99.5895**	**99.6002**	**99.6078**	**99.5880**	**99.5781**	**99.6223**
10	UACI	**33.3561**	**33.4246**	**33.4130**	**33.4110**	**33.3838**	**33.4363**	**33.5498**	**33.4906**	**33.4433**	**33.3561**
	NPCR	**99.6170**	**99.5872**	**99.5777**	**99.5884**	**99.6170**	**99.6048**	**99.5926**	**99.6140**	**99.5987**	**99.6170**

**Table 13:** Calculated UACI and NPCR for different combinations of *p* and *r* while *q* = 1 for Cameraman.

*p*/*r*		1	2	3	4	5	6	7	8	9	10
1	UACI	7.4904	2.6620	5.1333	11.4982	3.7695	5.6794	5.3902	11.9543	8.4184	7.4904
	NPCR	29.8447	10.6064	20.4529	45.8130	15.0192	22.6288	21.4767	47.6303	33.5419	29.8447
2	UACI	1.0214	0.1943	0.4910	1.9258	0.3592	0.5052	0.4911	1.8747	1.0055	1.0214
	NPCR	83.5632	49.5514	75.0793	86.7004	63.8062	76.2024	75.0595	84.4025	81.1432	83.5632
3	UACI	**33.7662**	**29.9298**	**32.1345**	**32.5679**	**33.5877**	**33.5277**	**33.6352**	**34.8690**	**33.6583**	**33.7662**
	NPCR	**99.6262**	**99.5071**	**99.5102**	**99.5285**	**99.5224**	**99.5850**	**99.6384**	**99.6552**	**99.6521**	**99.6262**
4	UACI	**33.4563**	**33.3303**	**33.4678**	**33.5979**	**33.4952**	**33.5201**	**33.4910**	**33.2905**	**33.3876**	**33.4563**
	NPCR	**99.6292**	**99.5453**	**99.5972**	**99.6582**	**99.5972**	**99.6170**	**99.5682**	**99.5743**	**99.6475**	**99.6292**
5	UACI	**33.2851**	**33.4706**	**33.3841**	**33.5766**	**33.3711**	**33.4932**	**33.3911**	**33.4998**	**33.4473**	**33.2851**
	NPCR	**99.6155**	**99.6262**	**99.5834**	**99.5972**	**99.6109**	**99.5956**	**99.5956**	**99.6368**	**99.6201**	**99.6155**
6	UACI	**33.4854**	**33.3612**	**33.6393**	**33.4269**	**33.3583**	**33.6404**	**33.4790**	**33.3590**	**33.2436**	**33.4854**
	NPCR	**99.6567**	**99.5911**	**99.5682**	**99.6078**	**99.6277**	**99.5880**	**99.6277**	**99.6124**	**99.6414**	**99.6567**
7	UACI	**33.4159**	**33.4013**	**33.5464**	**33.5039**	**33.4404**	**33.4768**	**33.4303**	**33.3951**	**33.2653**	**33.4159**
	NPCR	**99.6674**	**99.5850**	**99.6094**	**99.6262**	**99.5880**	**99.6140**	**99.5941**	**99.5850**	**99.6002**	**99.6674**
8	UACI	**33.4321**	**33.4036**	**33.6195**	**33.4753**	**33.3993**	**33.5394**	**33.4112**	**33.5314**	**33.4387**	**33.4321**
	NPCR	**99.5621**	**99.5926**	**99.6368**	**99.6277**	**99.5895**	**99.5941**	**99.5819**	**99.5987**	**99.6094**	**99.5621**
9	UACI	**33.4651**	**33.3438**	**33.4917**	**33.4837**	**33.4551**	**33.4780**	**33.4704**	**33.4806**	**33.4516**	**33.4651**
	NPCR	**99.6140**	**99.6323**	**99.5636**	**99.6216**	**99.6429**	**99.6109**	**99.6338**	**99.5758**	**99.6002**	**99.6140**
10	UACI	**33.6242**	**33.5821**	**33.2618**	**33.4312**	**33.5802**	**33.6133**	**33.3934**	**33.6385**	**33.5515**	**33.6242**
	NPCR	**99.5972**	**99.5895**	**99.6078**	**99.5773**	**99.6033**	**99.5911**	**99.6323**	**99.6109**	**99.5850**	**99.5972**

**Table 14:** Calculated UACI and NPCR for different combinations of *p* and *r* while *q* = 1 for Chess-plate.

*p*/*r*		1	2	3	4	5	6	7	8	9	10
1	UACI	49.7511	38.9683	0.6828	0.3194	17.8328	9.1234	1.8446	0.6682	24.8476	45.3508
	NPCR	99.1135	77.6321	2.7206	81.4575	71.0526	72.7020	14.6988	85.1974	99.0021	90.3473
2	UACI	30.8111	26.3993	34.1747	33.5065	33.3622	33.4134	33.0488	33.5821	33.5865	33.5562
	NPCR	72.5723	90.6128	99.3164	99.3210	99.5193	99.5987	99.4949	99.6674	99.5697	99.6399
3	UACI	**33.6698**	**33.2530**	**33.4078**	**33.4257**	**33.5033**	**33.4428**	**33.4594**	**33.5224**	**33.3517**	**33.4282**
	NPCR	**99.5483**	**99.6460**	**99.5850**	**99.5682**	**99.6063**	**99.6033**	**99.5819**	**99.6338**	**99.5972**	**99.6002**
4	UACI	**33.4568**	**33.4550**	**33.4542**	**33.4903**	**33.5600**	**33.3137**	**33.4928**	**33.5251**	**33.4225**	**33.3196**
	NPCR	**99.6185**	**99.6201**	**99.6262**	**99.5819**	**99.5850**	**99.5972**	**99.5895**	**99.6216**	**99.5956**	**99.6063**
5	UACI	**33.4745**	**33.5885**	**33.5112**	**33.5035**	**33.4013**	**33.4363**	**33.4302**	**33.5542**	**33.4494**	**33.4572**
	NPCR	**99.6155**	**99.6414**	**99.6292**	**99.6262**	**99.5850**	**99.5956**	**99.6063**	**99.6262**	**99.6109**	**99.6002**
6	UACI	**33.4729**	**33.4588**	**33.3505**	**33.5044**	**33.5310**	**33.5108**	**33.3838**	**33.6189**	**33.3733**	**33.4620**
	NPCR	**99.6277**	**99.5728**	**99.6292**	**99.5987**	**99.6048**	**99.6689**	**99.5865**	**99.6017**	**99.5941**	**99.6109**
7	UACI	**33.4701**	**33.4639**	**33.3825**	**33.5426**	**33.4590**	**33.4672**	**33.3788**	**33.4522**	**33.4046**	**33.4853**
	NPCR	**99.5926**	**99.6246**	**99.5758**	**99.6368**	**99.5941**	**99.6078**	**99.6201**	**99.6399**	**99.6048**	**99.5682**
8	UACI	**33.4681**	**33.4529**	**33.3931**	**33.3834**	**33.6822**	**33.4259**	**33.3729**	**33.3856**	**33.4834**	**33.4206**
	NPCR	**99.5926**	**99.6811**	**99.6063**	**99.5941**	**99.5972**	**99.6002**	**99.6643**	**99.6368**	**99.5987**	**99.6155**
9	UACI	**33.3928**	**33.5164**	**33.5825**	**33.4455**	**33.5033**	**33.4134**	**33.4258**	**33.5132**	**33.5198**	**33.6166**
	NPCR	**99.5956**	**99.6231**	**99.6078**	**99.6216**	**99.6262**	**99.6094**	**99.6033**	**99.6094**	**99.6216**	**99.6292**
10	UACI	**33.2732**	**33.5652**	**33.5405**	**33.6319**	**33.3404**	**33.4587**	**33.3828**	**33.4207**	**33.4494**	**33.5986**
	NPCR	**99.5911**	**99.5895**	**99.6185**	**99.6002**	**99.6078**	**99.6063**	**99.6231**	**99.6216**	**99.5590**	**99.6536**

**Table 15:** Results of security analysis.

Image name	*p*	*q*	*r*	Plain entropy	Cipher entropy	Plain image correlations			Cipher image correlations			UACI	NPCR
						HC	VC	DC	HC	VC	DC		
Cameraman 256 × 256	1	1	1	7.0097	7.9969	0.8390	0.7189	0.6973	0.0003	0.0012	0.0013	7.4904	29.8447
	3	1	1		7.9976				0.0057	−0.0049	0.0027	33.7662	99.6262
	1	3	1		7.9971				0.0013	0.0035	−0.0030	7.4904	29.8447
	1	1	3		7.9972				0.0011	0.0011	−0.0042	5.1333	20.4529

Chess-plate 256 × 256	1	1	1	1	7.9970	0.9775	0.9800	0.9637	−0.0096	−0.0056	0.0056	49.7511	99.1135
	3	1	1		7.9974				0.0193	−0.0231	0.0048	33.6698	99.5483
	1	3	1		7.9972				−0.0010	0.0102	0.0111	49.7511	99.1135
	1	1	3		7.9973				0.0123	−0.0053	0.0206	0.6828	2.7206

Baboon 512 × 512	1	1	1	7.3579	7.9993	0.8644	0.7587	0.7261	−0.0038	0.0033	0.0015	34.5764	68.8828
	3	1	1		7.9993				0.0015	−0.0004	0.0009	33.4245	99.6040
	1	3	1		7.9993				0.0012	−0.0004	−0.0007	34.5764	68.8828
	1	1	3		7.9993				0.0016	0.0018	−0.0024	1.9700	3.9246

Peppers 512 × 512	1	1	1	7.5714	7.9993	0.8642	0.7587	0.7261	−0.0030	0.0018	−0.0017	0.1867	47.6059
	3	1	1		7.9993				−0.0047	−0.0032	−0.0009	33.9480	99.3217
	1	3	1		7.9993				0.0005	0.0007	0.0012	0.1867	47.6059
	1	1	3		7.9993				−0.0008	−0.0025	−.0009	11.3506	90.4499

Fingerprint 512 × 512	1	1	1	6.7279	7.9993	0.8644	0.7587	0.7261	0.0040	−0.0010	0.0049	0.1102	14.0533
	3	1	1		7.9992				−0.0009	0.0009	−0.0032	32.7711	99.6140
	1	3	1		7.9994				−0.0014	−0.0002	−0.0013	0.1102	14.0533
	1	1	3		7.9993				0.0043	−0.0007	−0.0007	0.2429	30.9715
